# Efficacy of the eHealth application Oncokompas, facilitating incurably ill cancer patients to self-manage their palliative care needs: A randomized controlled trial

**DOI:** 10.1016/j.lanepe.2022.100390

**Published:** 2022-04-21

**Authors:** Anouk S. Schuit, Karen Holtmaat, Birgit I. Lissenberg-Witte, Simone E.J. Eerenstein, Josée M. Zijlstra, Corien Eeltink, Annemarie Becker-Commissaris, Lia van Zuylen, Myra E. van Linde, C. Willemien Menke-van der Houven van Oordt, Dirkje W. Sommeijer, Nol Verbeek, Koop Bosscha, Rishi Nandoe Tewarie, Robert-Jan Sedee, Remco de Bree, Alexander de Graeff, Filip de Vos, Pim Cuijpers, Irma M. Verdonck-de Leeuw

**Affiliations:** a Department of Clinical, Neuro and Developmental Psychology, Faculty of Behavioral and Movement Sciences, Amsterdam Public Health Research Institute, Vrije Universiteit Amsterdam, the Netherlands; b Cancer Center Amsterdam (CCA), Amsterdam UMC, Vrije Universiteit Amsterdam, Amsterdam, the Netherlands; c Amsterdam UMC, Vrije Universiteit Amsterdam, Epidemiology and Data Science, Amsterdam, the Netherlands; d Amsterdam UMC, Vrije Universiteit Amsterdam, Otolaryngology – Head and Neck Surgery, Cancer Center Amsterdam, Amsterdam, the Netherlands; e Department of Hematology, Amsterdam UMC, Vrije Universiteit Amsterdam, Cancer Center Amsterdam, Amsterdam, the Netherlands; f Department of Pulmonary Diseases, Amsterdam UMC, Vrije Universiteit Amsterdam, Cancer Center Amsterdam, Amsterdam, the Netherlands; g Department of Medical Oncology, Amsterdam UMC, Vrije Universiteit Amsterdam, Cancer Center Amsterdam, Amsterdam, the Netherlands; h Department of Internal Medicine, Flevo Hospital, Almere, the Netherlands; i Department of Oncology, St. Antonius hospital, Utrecht, the Netherlands.; j Department of Surgery, Jeroen Bosch hospital, Den Bosch, the Netherlands; k Department of Neurosurgery, Haaglanden MC, The Hague, the Netherlands; l Department of Otolaryngology, Head and Neck Surgery, Haaglanden MC, The Hague, the Netherlands; m Department of Head and Neck Surgical Oncology, University Medical Center Utrecht, University Utrecht, the Netherlands; n Department of Medical Oncology, Cancer Center, University Medical Center Utrecht, University Utrecht, the Netherlands

**Keywords:** eHealth, Palliative care, Supportive care, Incurable cancer, Psychosocial oncology

## Abstract

**Background:**

Many patients with incurable cancer have symptoms affecting their health-related quality of life. The eHealth application ‘Oncokompas’ supports patients to take an active role in managing their palliative care needs, to reduce symptoms and improve health-related quality of life (HRQOL). This randomized controlled trial was conducted to determine the efficacy of Oncokompas compared to care as usual among incurably ill cancer patients with a life expectancy of more than three months.

**Methods:**

Patients were recruited in six hospitals in the Netherlands. Eligible patients were randomly assigned to the intervention (direct access to Oncokompas) or the control group (access to Oncokompas after three months). The primary outcome measure was patient activation (i.e., patients’ knowledge, skills and confidence for self-management). Secondary outcomes were general self-efficacy and HRQOL. Measures were assessed at baseline, two weeks after randomization, and three months after the baseline measurement. Linear mixed models were used to compare longitudinal changes between both groups from baseline to the three-month follow-up.

**Findings:**

In total, 219 patients were eligible of which 138 patients completed the baseline questionnaire (response rate 63%), and were randomized to the intervention (69) or control group (69). There were no significant differences between the intervention and control group over time in patient activation (estimated difference in change T0-T2; 1·8 (90% CI: -1·0 to 4·7)), neither in general self-efficacy and HRQOL. Of the patients in the intervention group who activated their account, 74% used Oncokompas as intended. The course of patient activation, general self-efficacy, and HRQOL was not significantly different between patients who used Oncokompas as intended versus those who did not.

**Interpretation:**

Among incurably ill cancer patients with a life expectancy of more than three months and recruited in the hospital setting, Oncokompas did not significantly improve patient activation, self-efficacy, or HRQOL.

**Funding:**

ZonMw, Netherlands Organization for Health Research and Development (844001105).

**Research in context**

**Evidence before this study**

Incurably ill cancer patients have to deal with physical, psychological, social, and existential symptoms related to cancer and its treatment. Palliative care is increasingly recognized as an integral part of cancer care. Also, there is growing interest in self-management and behavioral intervention technologies to improve (access to) palliative care. Evidence on the effects of these interventions in palliative care is promising but limited. The application Oncokompas was developed to monitor physical, psychological, social and existential domains of quality of life, to provide personalized information on quality of life, and to support cancer patients to adopt an active role in managing their disease, adjusted to their personal well-being and preferences. Several studies were conducted to examine the effects of Oncokompas among cancer survivors, showing promising effects on HRQOL but limited effect on patient activation. The current study is conducted to investigate efficacy of Oncokompas among incurably ill cancer patients.

**Added value of this study**

The findings of this randomized controlled trial show that Oncokompas is not effective to improve patient activation among incurably ill cancer patients with a life expectancy of more than three months and recruited in the hospital setting. Also, no effects were found on self-efficacy and health-related quality of life (HRQOL). This RCT contributes to knowledge on the effects and usage of behavioral intervention technologies in palliative cancer care.

**Implications of all the available evidence**

To support cancer patients to take an active role in managing their disease and healthcare, it is important to facilitate the uptake of self-management behaviors. Offering patients access to fully automated and self-guided eHealth interventions such as Oncokompas, might help to create a shift in patients’ self-management behavior. The lack of effect of Oncokompas in our study may be due to the relatively good performance of the included patients on the outcome measures at baseline or that Oncokompas in its current form needs more tailoring to incurably ill cancer patients. Research on the possibilities to further personalize behavioral intervention technologies is needed, to create an optimal fit between intervention technologies and patients’ needs. Future research on efficacy of behavioral intervention technologies, such as Oncokompas, that aim to improve HRQOL, should include users who have a need for palliative care.

Alt-text: Unlabelled box

## Introduction

Incurable cancer challenges patients to deal with physical, psychological and social symptoms, and existential concerns, affecting aspects of their health-related quality of life (HRQOL).^1^^,^^2^ Maintaining optimal HRQOL by early identification of symptoms and providing access to palliative care services if needed, is an important aspect of palliative care.

Many cancer patients want to be in charge of their own life as long as possible. Moreover, there is a growing demand on healthcare resources and patients are increasingly expected to adopt an active role in managing their illness and well-being.^3^ The tasks that people undertake to deal with managing their health are referred to as *self-management*.^4^ Self-management strategies are dependent on individual preferences and characteristics and cover multiple domains,^5^ including monitoring symptoms and treatment effects, adjusting nutrition and diet, maintaining daily routine by adjusting daily activities, and seeking social support.^5^

Interventions to support self-management are becoming an integral component of care and can have positive effects in cancer patients.^6^ Furthermore, eHealth interventions are available to detect and manage side effects of cancer and its treatment.^7^ These interventions enable patients to be actively engaged in healthcare, improve health outcomes, and lead to positive behavior change.^8^^,^^9^ Earlier research suggests that if patients’ activation level is increased, improved self-management behaviors will follow.^10^ Activated patients – with knowledge, skills and confidence for self-management^11^ – function as collaborative partners in managing their health.^10^^,^^11^ Previous studies reported positive effects of eHealth interventions on HRQOL and described the ability of eHealth to track symptoms over time, access web-based information, and provide prompts when to contact healthcare professionals.^6^

The eHealth application Oncokompas was developed to support cancer patients to adopt an active role to self-manage their symptoms and improve their well-being. Oncokompas is a behavioral intervention technology (BIT), which is – as described by Mohr et al. – an application which uses features of information and communication technology aimed at changing behavioral and mental health outcomes.^12^ Oncokompas is meant as additional support for cancer patients and is based on the stepped care principle, supporting patients to take actions to deal with their symptoms by themselves, and with professional guidance if needed. By using Oncokompas, patients can monitor their symptoms using Patient-Reported Outcome Measures (PROMs) and get feedback and advice, supporting them to deal with symptoms by themselves. Patients also get an overview of supportive care options where they can go to when self-care is not sufficient and professional care is needed.^13^^,^^14^ Patients can use Oncokompas at their own pace, with 24/7 availability. Initially, Oncokompas was developed targeting cancer survivors.^13^ Research showed that using Oncokompas improves HRQOL and reduces symptoms among survivors,^14^ and is as cost-effective as usual care.^15^

The content of the application was extended for use among patients with incurable cancer. A pilot study on the feasibility of self-management support delivered by nurses in the home setting, with Oncokompas integrated as eHealth component, showed that incurably ill cancer patients positively assessed Oncokompas as a self-management intervention. However, usage of the intervention was low and Oncokompas had no significant effect on patient activation or HRQOL, which may be explained by the fact that many pilot participants were already very ill (near the end-of-life), and that the self-management support delivered by nurses was superior to the eHealth application.^16^ Based on these findings, it was hypothesized that Oncokompas may be more beneficial in patients with longer life expectancy regarding patient activation and HRQOL, and as fully automated behavioral intervention technology.

The aim of this study was to determine the efficacy of the eHealth self-management application Oncokompas as BIT additional to care as usual and compared to care as usual only, on patient activation, general self-efficacy, and HRQOL among incurably ill cancer patients, who have a life expectancy of at least three months. The hypothesis is that Oncokompas supports incurably ill cancer patients to improve their knowledge, skills and confidence to self-manage their symptoms and improve their well-being.

## Methods

### Study design

This prospective randomized controlled trial with two parallel groups targeted incurably ill cancer patients. Patients in the intervention group got access to Oncokompas directly after completing the baseline questionnaire and patients in the control group after three months (i.e., after completing the last questionnaire). Outcome measures were collected through an online questionnaire at baseline (t0), two weeks after randomization (t1) and three months after the baseline measurement (t2).

The study protocol was approved by the Medical Ethics Committee of VU University Medical Center (2018.224). All participants provided written informed consent. The study protocol was published previously.^17^ This trial was registered in the Netherlands Trial Register (NTR 7494/NL7285). The CONSORT guidelines (CONsolidated Standards of Reporting Trials) were used to report on the results of this trial.^18^

### Study population

Inclusion criteria were: (1) being diagnosed with incurable cancer (not having curative treatment options), (2) having a life expectancy of at least three months (not being in the end-of-life phase of cancer), and (3) being aware of the cancer's incurability. Patients were excluded if (1) they had severe cognitive impairments, (2) they had poor understanding of the Dutch language (not able to complete Dutch questionnaires), (3) they were too ill to participate, (4) they did not have access to the Internet or to an e-mail account, (5) their healthcare professional thought that participation would be too burdensome due to the patient's participation in other studies, or (6) they already used Oncokompas before (in previous research).

### Study procedures

Eligible patients were informed about the study by their physician, nurse or nurse specialist, at six hospitals in the Netherlands (Amsterdam University Medical Centers, University Medical Center Utrecht, St. Antonius Hospital, Haaglanden Medical Center, and Jeroen Bosch Hospital). When patients were interested, their healthcare provider asked permission to share their contact details with the researchers of the Vrije Universiteit Amsterdam (VU). Interested patients were then contacted by phone by the researcher to receive more information about the study. After signing informed consent, patients received the first questionnaire by e-mail. Thus, patients were informed on the study by their health care professional from the hospital and included in the study by the research team of the VU.

### Care as usual

All patients received care as usual (CAU) during their study participation. CAU was defined as the care provided by the oncological team or other healthcare professionals, including all medical and supportive care that patients receive, regardless of study participation.

### Intervention

Oncokompas is an eHealth self-management application, consisting of three steps: measure, learn and act. Screenshots of Oncokompas and an overview of the topics covered within Oncokompas can be found in the supplementary material. Patients logging in to Oncokompas first enter the step ‘Measure’, where they complete a general questionnaire used to select the topics appropriate for this patient (e.g., when someone is retired, the topic about ‘work’ will not be shown). Then patients can select which topics they want to address within Oncokompas. Subsequently, PROMs are used to monitor patients’ physical, psychological, social and existential well-being. In the next step, ‘Learn’, Oncokompas provides information and feedback on patients’ outcomes, tailored to their health status, personal characteristics and preferences. Using a traffic-light system (green, orange and red), patients get an overview of their overall well-being on topic level. A green score means that the patient is doing well on this topic, an orange score means that this topic *could* use attention and support, and a red score means that this topic *needs* attention and support. Then, Oncokompas provides comprehensive self-care advice, such as tips and tools. Lastly, within the step ‘Act’, patients receive a personal overview of supportive care options, with options for professional guidance when needed.

Oncokompas was developed using a stepwise, iterative and participatory approach, actively involving end users and oncological and palliative health care health professionals in the design process.^19^

More information about Oncokompas is available in the study protocol.^17^

### Randomization

Patients were randomly assigned to the intervention or control group (1:1 ratio), using block randomization. Stratification was not applied. The randomization scheme was a computer-generated table with random numbers (with a random block length of four, six or eight), created by a researcher not involved in the study, who also performed the allocation of participants. Neither the participants nor the coordinating researcher were blinded after assignment to the intervention, due to the nature of the study intervention.

### Study measures

Since Oncokompas primarily aims to stimulate self-management, the primary outcome measure of the study was patient activation, measured with the Patient Activation Measure (PAM), a widely recognized questionnaire to measure self-management abilities.^20^ The PAM measures patients’ self-reported knowledge, skills and confidence for self-management of their health or chronic condition.^11^ It consists of 13 items with a 4-point Likert scale (i.e., strongly disagree, disagree, agree, and strongly agree) and the option “not applicable”. Some items are for example: “Taking an active role in my own healthcare is the most important factor in determining my health and ability to function” and “I know what each of my prescribed medications do”. The total PAM score ranges from 0 up to 100 and is computed by calculating the mean score of all applicable items, which is transformed to a standardized activation score (non-applicable items are not taken into account). Scores can be divided into four levels, ranging from low activation to high activation. A higher total PAM score indicates a higher level of patient activation. A difference of four points on the PAM is considered to be clinically meaningful.^21^^,^^22^

Secondary outcome measures were self-efficacy and HRQOL. The General Self-Efficacy Scale (GSE) assesses how a person deals with difficult situations in life,^23^ consisting of ten items with a 4-point Likert scale (not at all true, hardly true, moderately true, and exactly true). There is no cut-off score available on the GSE; the international average for the GSE sum score is 29.55.^24^ Higher GSE scores indicate higher self-efficacy.^23^ The total score ranges from 10 to 40, calculated by adding up the scores on all items, as long as no more than three items are missing.

The European Organization for Research and Treatment of Cancer Quality of Life Questionnaire for patients in palliative care (EORTC-QLQ-C15-PAL) was used to measure (domains of) HRQOL^25^ and consists of 15 items. The questionnaire includes a global quality of life scale, two functional scales (physical and emotional functioning), two symptom scales (fatigue and pain), and five symptom scales based on single items (nausea, dyspnea, insomnia, appetite loss, and constipation). Subscale scores range from 0 up to 100. Higher scores on the global quality of life scale and functional scales represent better HRQOL, while higher scores on symptom scales indicate higher levels of symptoms. Studies regarding the minimal clinically important differences (MCIDs) for the EORTC-QLQ-C15-PAL are limited and inconclusive.^26^

Patients’ sociodemographic and clinical characteristics were assessed at baseline using a study specific questionnaire. Information on patients’ cancer type and treatment modality were retrieved from medical files.

### Sample size

To demonstrate an increase of at least 0·5 standard deviations in the intervention group compared to the control group (i.e., between group change of 0·5 SD) on the PAM between t0 and t2 as statistically significant in a one-tailed test using a power of 80% (1-β = 0·80) and a significance level of 5% (α=0·05), 51 participants were required in each study arm at three-months follow-up. Anticipating a dropout rate of 25% between t0 and t2, the aim was to include 136 patients; 68 participants per study arm at baseline.

### Statistical analyses

Descriptive statistics were generated to compare sociodemographic characteristics, clinical characteristics and outcome measures at baseline between the intervention group and control group.

Linear Mixed Models (LMM) were used to compare longitudinal changes in primary and secondary outcome measures in both study arms between t0, t1, and t2. Fixed effects were used for study arm, measurement, and their two-way interaction, and a random intercept for subjects. Missing data was not imputed as LMM accounts for missing data.

This RCT was conducted partly before and partly during the COVID-19 pandemic. Additional analyses were performed to analyze a possible effect of the pandemic, using LMM (measurement * group * (time of participation)). A categorical variable was created representing three groups: patients who participated before COVID-19 pandemic (cut-off date set at 12 March 2020, when the Dutch government advised all citizens to stay at home),^27^ patients who were included before the pandemic but completed follow-up measurements during the pandemic, and patients who were included during the pandemic.

Furthermore, to analyze a possible effect of how Oncokompas was used, Oncokompas’ logging data of users were used. Usage as intended was defined as completion of the components ‘Measure’ and ‘Learn’ for at least one topic. Additional LMM analyses were performed to analyze a possible effect of usage (measurement * usage). Univariable logistic regression models were used to examine whether outcome measures, sociodemographic and clinical characteristics predicted usage as intended.

All analyses were performed using IBM Statistical Package for the Social Sciences (SPSS) version 26 (IBM Corp., Armonk, NY USA) and according to the intention-to-treat principle. A *p*-value of < 0·05 was considered significant for all analyses.

### Role of the funding source

The funder of the study had no role in study design, data collection, data analysis, data interpretation, or writing the report.

## Results

### Study population

**Figure 1:**
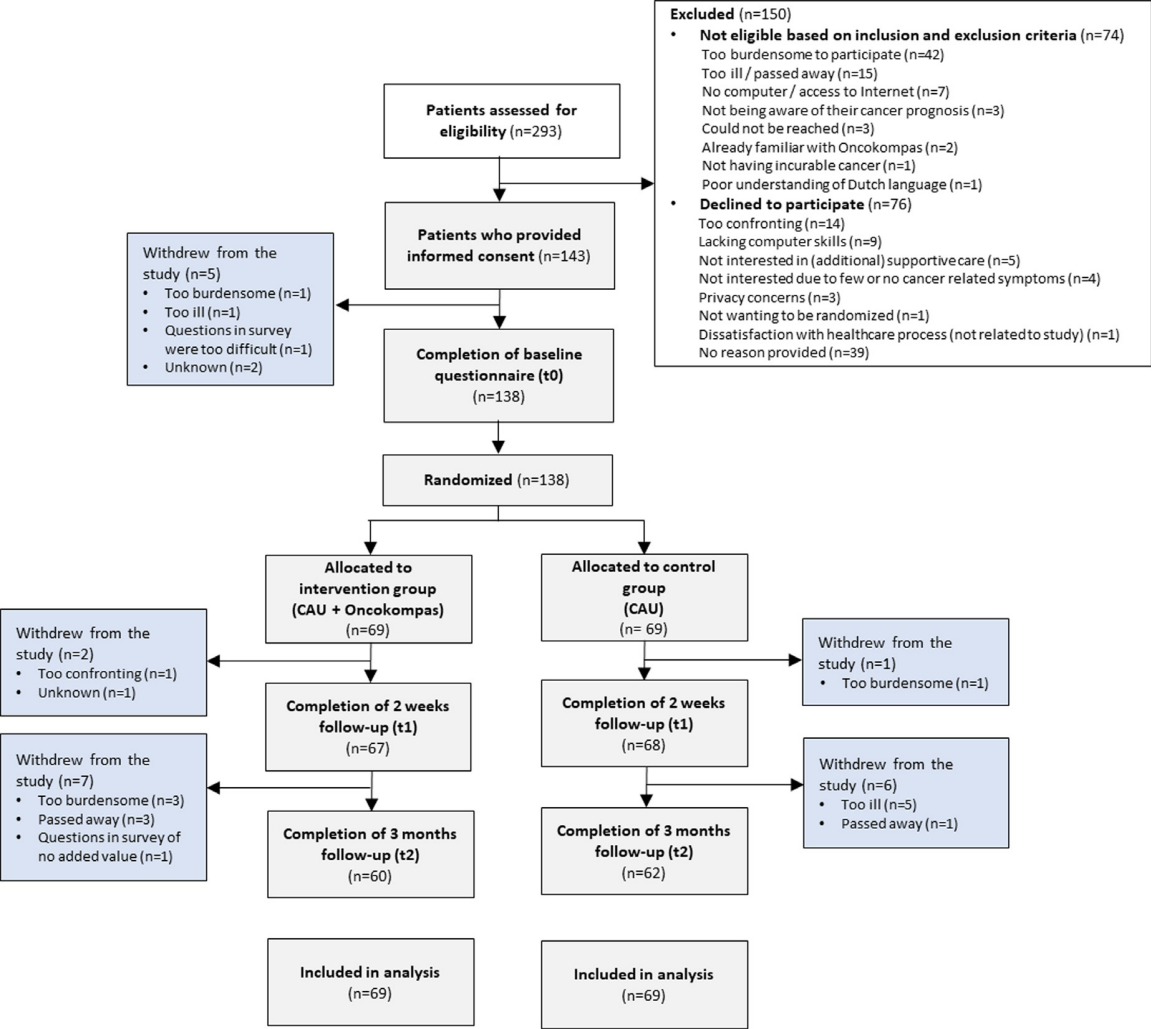
Flow diagram of the study.

From December 13, 2018 to August 27, 2020, 293 patients were referred to the research team, of whom 219 were eligible for inclusion. In total, 143 patients signed informed consent, of which 5 patients declined participation upon receiving the baseline questionnaire and were not included (response rate 63%). Reasons for declining participation were: participation being too confronting (*n* = 14), lacking computer skills (*n* = 9), not being interested (*n* = 9), privacy concerns (*n* = 3), and other reasons (*n* = 5); 41 patients provided no reason for non-participation (Figure 1). In total, 62 patients were included before the COVID-19 pandemic (of which 25 patients participated partly during the pandemic) and 76 patients during the pandemic.

In total, 138 patients completed the baseline questionnaire, of which 69 patients were allocated to the intervention and 69 to the control group. Gender balance was achieved and the majority had a partner (83%). A large group of participants were highly educated (47%), were diagnosed with brain tumors (28%), and received at least one type of treatment during study participation (91%) (Table 1).

**Table 1:** Sociodemographic and clinical characteristics of the study participants at baseline.

	Control group (*n* = 69)		Intervention group (*n* = 69)		Total group (*n* = 138)	
	Number	%	Number	%	Number	%
Age in years						
Mean (SD)	62·3 (11·9)	-	60·0 (12·7)	-	61·1 (12·3)	-
25th-75th percentile	54·5 – 71·5	-	51 – 68·5	-	53 – 70·3	-
Sex						
Male	37	54	37	54	74	54
Female	32	46	32	46	64	46
Education level^a^						
Low	19	28	19	28	38	28
Medium	18	26	16	23	34	25
High	31	45	34	49	65	47
Other/unknown	1	1	-	-	1	1
Marital status, partner						
Yes	57	83	58	84	115	83
No	12	17	11	16	23	17
Children						
Yes	54	78	52	75	106	77
No	15	22	17	25	32	23
Employed						
Yes	28	41	23	33	51	37
No	41	59·4	46	67	87	63
Tumor type						
Lung cancer	8	12	8	12	16	12
Hematological cancer	8	12	8	12	16	12
Brain tumor	22	32	17	25	39	28
Head and neck cancer	7	10	9	13	16	12
Breast cancer	5	7	10	15	15	11
Gastro-intestinal cancer	10	15	9	13	19	14
Urological cancer	6	9	4	6	10	7
Other	1	1	3	4	4	3
Multiple primaries^b^	2	3	1	1	3	2
Anti-cancer treatment						
None	7	10	5	7	12	9
Single treatment	49	71	49	71	98	71
Combination or multimodal treatment	13	19	15	22	28	20
Comorbidities						
No comorbidities	37	54	28	41	65	47
One comorbidity	17	25	22	32	39	28
Two or more comorbidities	15	22	19	28	34	25

a Low = elementary school/preparatory secondary vocational education (VMBO), Middle = secondary vocational education (MBO)/general secondary education (HAVO)/pre-university education (VWO), High = higher vocational education (HBO)/university (WO). Dutch abbreviations of the school types are specified between the brackets.

b Three patients were diagnosed with multiple primary tumors and therefore shown in a separate category.

### Efficacy of Oncokompas on patient activation, general self-efficacy, and HRQOL

The results of the linear mixed model analyses are shown in Table 2. No significant differences were found in the course of patient activation over time in the intervention group compared to the control group (estimated difference in change T0-T2; 1·8 (90% CI -1·0 to 4·7); *p*-value two-way interaction = 0·56).

**Table 2:** Mean scores per group per assessment and results of the linear mixed model analyses on primary and secondary outcome measures for the total group.

	Baseline (t0)		2 weeks follow-up (t1)		3-months follow-up (t2)			
	N	Mean (SD)	N	Mean (SD)	N	Mean (SD)	Estimated difference in change between T0 and T2 (90% CI)	P-value two-way interaction
Patient activation (PAM)								0·56
Intervention	65	55·6 (11·5)	65	55·1 (12·5)	59	56·4 (11·7)	1·8 (-1·0 to 4·7)	
Control	68	55·1 (11·5)	68	54·7 (9·8)	61	54·7 (11·6)		
General self-efficacy (GSE)								0·23
Intervention	69	29·5 (5·5)	67	29·5 (5·4)	60	30·0 (5·7)	1·0 (-0·2 to 2·2)	
Control	69	31·1 (4·5)	68	29·9 (4·6)	62	30·6 (4·3)		
HRQOL (EORTC-QLQ-C15-PAL)								
Global quality of life								0·69
Intervention	69	73·9 (18·8)	67	69·9 (18·2)	60	70·3 (21·3)	-2·6 (-7·5 to 2·4)	
Control	69	73·4 (18·4)	68	70·6 (22·5)	62	72·6 (18·9)		
Physical functioning								0·23
Intervention	69	88·1 (14·4)	67	88·2 (13·4)	60	87·0 (15·2)	-0·8 (-4·3 to 2·6)	
Control	69	90·3 (16·3)	68	87·6 (18·5)	62	88·7 (18·4)		
Emotional functioning								0·32
Intervention	69	71·7 (24·3)	67	74·9 (22·4)	60	71·9 (26·3)	4·6 (-1·0 to 10·1)	
Control	69	81·2 (21·0)	68	81·1 (20·7)	62	77·7 (22·2)		
Fatigue								0·27
Intervention	69	45·4 (28·0)	67	42·0 (24·7)	60	44·4 (28·6)	-3·2 (-9·8 to 3·4)	
Control	69	35·7 (25·6)	68	39·0 (30·1)	62	37·9 (27·7)		
Pain								0·54
Intervention	69	27·5 (28·1)	67	27·9 (24·3)	60	29·4 (27·3)	2·6 (-3·7 to 8·9)	
Control	69	22·9 (25·3)	68	25·2 (26·5)	62	24·2 (27·1)		
Dyspnea								0·32
Intervention	69	19·3 (23·2)	67	22·4 (25·5)	60	21·1 (25·3)	4·7(-1·3 to 10·6)	
Control	69	19·3 (25·8)	68	17·6 (26·7)	62	15·6 (25·4)		
Insomnia								0·91
Intervention	69	33·3 (31·8)	67	31·3 (30·6)	60	32·2 (28·1)	0·4 (-7·1 to 7·9)	
Control	69	29·5 (30·0)	68	28·9 (28·7)	62	25·8 (29·8)		
Appetite loss								0·66
Intervention	69	22·2 (30·6)	67	19·4 (30·2)	60	21·7 (29·3)	-2·3 (-10·2 to 5·6)	
Control	69	18·4 (26·5)	68	20·1 (29·4)	62	22·0 (29·5)		
Nausea								0·68
Intervention	69	18·8 (30·0)	67	13·9 (24·0)	60	20·6 (28·2)	2·8 (-5·6 to 11·2)	
Control	69	18·8 (24·6)	68	15·7 (27·3)	62	18·3 (26·8)		
Constipation								0·48
Intervention	69	24·2 (27·3)	67	20·9 (25·8)	60	22·2 (26·5)	-5·8 (-14·0 to 2·3)	
Control	69	18·8 (21·8)	68	19·1 (27·8)	62	21·5 (29·0)		

Also, the course of general self-efficacy did not differ significantly between patients in the intervention and control group (1·0 (-0·2 to 2·2); *p*-value two-way interaction = 0·23), nor the course of HRQOL (all domains) (Table 2, *p*-values of two-way interactions ranging from 0·23 to 0·91).

### Usage of Oncokompas

Of the 69 patients in the intervention group, 65 activated their account and 48 of them (74%) used Oncokompas as intended during the three-month follow-up period. The median number of logins among intended users was 3 (interquartile range (IQR) = 2·0 - 4·0). Topics that were most often chosen were: coping with emotions (*n* = 17), cancer related anxiety (*n* = 12), side-effects of medical treatment (*n* = 12), fatigue (*n* = 10), tenseness (*n* = 9), depression (*n* = 8), and body weight (*n* = 8).

The course of patient activation (-1·2 (90% CI: -5·8 to 3·5); *p*-value two-way interaction = 0·91), general self-efficacy (1·2 (90% CI: -0·7 to 3·2); *p*-value two-way interaction = 0·49), and HRQOL (Supplementary material) all domains *p*-values two-way interactions ranging from 0·081 to 0·92) was not significantly different between patients who used Oncokompas as intended versus those who did not (Supplementary material).

### COVID-19 pandemic

The efficacy of the intervention was not significantly influenced by the COVID-19 pandemic regarding patient activation (*p*-value three-way interaction = 0·056) and general self-efficacy (*p*-value three-way interaction = 0·063) (Supplementary material; Table 3). There was an effect on the HRQOL subscale dyspnea (*p*-value three-way interaction = 0·018). Patients included during the pandemic showed small differences in the course of dyspnea over time (Supplementary material; Figure 2). Among patients who were included before the COVID-19 pandemic and completed their follow-up during the pandemic, the course of dyspnea was better in the intervention group than in the control group at three-months follow-up (Supplementary material; Figure 2). Participation before or during the COVID-19 pandemic did not moderate other HRQOL domains (*p*-values three-way interactions ranging from 0·14 to 0·94).

## Discussion

This RCT investigated the efficacy of the eHealth application Oncokompas showed no significant improvements on patient activation, self-efficacy, or HRQOL among incurably ill cancer patients with a life expectancy of more than three months.

Previous studies showed that eHealth applications can positively affect patient empowerment in palliative care and contribute to efficient use of palliative care resources.^28^ However, effects on HRQOL are inconclusive.^29^ In this study, no effects were found on patient activation, similar to another RCT among cancer survivors.^14^ Furthermore, Oncokompas did not improve (different domains of) HRQOL among incurably ill cancer patients, similar to a previous RCT on Oncokompas among colon cancer survivors.^30^ In contrast, the RCT among cancer survivors (breast-, colorectal-, head and neck cancer, and lymphoma), demonstrated that Oncokompas was beneficial to improve HRQOL (small effect size) and to reduce tumor-specific symptoms (larger effect sizes).^14^ There may have been a ceiling effect of Oncokompas' effects on HRQOL, since HRQOL of participants in all these studies was already high at baseline (mean summary score of the EORTC QLQ-C30 was 87·4 among colon cancer survivors^30^ and 85·3 among various cancer survivors^14^). Mean global quality of life score on the EORTC QLQ-C15 PAL among participants in this study was 73·9 (the EORTC QLQ-C15 PAL does not contain a summary score as the QLQ-C30).

A qualitative study was conducted alongside the RCT to obtain insight in patients’ self-management strategies to cope with cancer and their experiences with Oncokompas. Interviews among cancer survivors and incurably ill cancer patients showed that objectives of self-management interventions like Oncokompas correspond well with strategies to cope with cancer, i.e. taking a certain responsibility for your well-being, and obtaining information and tailored supportive care options.^31^ Due to differences in informational preferences during the cancer trajectory, and varying informational needs, eHealth solutions should be customizable to individual patients’ needs.^32^ Benefits from Oncokompas among cancer survivors were largely gained because of tumor-specific topics.^14^ In the present study, Oncokompas was adapted to the needs of incurably ill patients in general and no tumor-specific topics were included. It may be that the application in its current form is not tailored enough. However, cancer-generic topics that were chosen frequently are similar among cancer survivors and incurably ill patients (fatigue and stress/tenseness).^33^

In the current study, 26% of the patients did not use Oncokompas as intended, which might have affected its efficacy. Reasons for not using Oncokompas were investigated in earlier studies: no symptom burden, a busy daily schedule, concentration problems, or having technical issues.^31^^,^^33^ To overcome the last two reasons, the interface design may be improved to easily navigate through the application and interactive and user-friendly multimedia formats could be added to present information. To stimulate self-management among patients, patients need to be prepared to actively manage their care and be engaged in a collaborative and empowering relationship with their healthcare professional. It might be helpful to train healthcare providers to support self-management, using techniques like motivational interviewing.^34^

A strength of this study is the high follow-up rate. A study limitation is that the study was not powered to examine the efficacy of Oncokompas among patients with different patient activation levels or HRQOL profiles. Analyses were performed to explore differences in the course between patients who used Oncokompas as intended versus those who did not. Those results should be interpreted with caution; these analyses were performed post hoc and it is not possible to interpret these findings in terms of causal relations. Additionally, the sample size of these groups was limited, leading to high uncertainty and imprecision of the findings. Incurably ill patients were included with a life expectancy of *at least* three months, and no upper limit. This may have resulted in a mixed study population regarding stressors and care needs, and might have affected the results. Oncokompas proved to be more effective among cancer survivors reporting a high burden of tumor-specific symptoms,^14^ which makes sense. In contrast, among incurably ill patients, cognitive problems may hamper usage and effectiveness of self-management applications. Since evidence on MCIDs was limited, it was not possible explain all results in terms of clinical importance, which may be concerned as a study limitation. Another limitation is that – due to privacy regulations – no information was collected of patients not interested in study participation. Also, no medical information was collected about the time since the start of the palliative phase, which could have been interesting to gain knowledge on how and when to implement behavioral intervention technologies for specific patient groups by examining the efficacy of these interventions among patients being aware of the incurability of their illness for a longer period of time versus patients who just found out. Lastly, the results of the secondary analyses should be interpreted with caution; the significant effects in these analyses could be explained due to multiple testing.

Future research investigating the effect of eHealth self-management interventions on patient activation and HRQOL, should specifically focus on cancer patients and survivors with low activation levels, impaired HRQOL or who express a need for supportive care. Furthermore, a longer follow-up might be necessary to detect changes in patient activation levels; it might take longer than three months’ time to develop self-management skills. Future studies should also include outcome measures to assess patients’ care needs in order to clarify the relationship between needs and usage on the application's efficacy. Additionally, it might be interesting to further examine usage of Oncokompas through logging data and evaluation forms, generating additional knowledge on topics of interest, informational preferences, and applicability in patients’ daily life.^33^ It would be interesting to investigate the accuracy of the current definition of usage as intended and to explore whether the relationship between efficacy and usage as intended is properly reflected.

Since Oncokompas is also available for partners of incurably ill patients,^35^ future research may investigate the effects of Oncokompas when dyads use the application together. Furthermore, 73% of the patients participated in this RCT during the COVID-19 pandemic, which might have influenced routine palliative care.^36^ Contacts with cancer patients may have changed from face-to-face contact to video consults, influencing the results. The results regarding dyspnea are puzzling and may be a coincidental finding due to multiple testing.

In conclusion, Oncokompas in its current format does not increase patient activation, general self-efficacy or HRQOL among incurably ill cancer patients with a life expectancy of more than three months. More insight is needed in the associations between care needs, usage and efficacy of behavioral intervention technologies such as Oncokompas, and the added value of further tailoring interventions to individual supportive care needs, to create an optimal fit between intervention technologies and patients’ needs.

## Declaration of interests

IVdL reports grants from the Netherlands Organization for Health Research and Development (ZonMw), the Dutch Cancer Society (KWF Kankerbestrijding), Bristol Myers Squibb, Danone Ecofund/Nutricia. ABC reports grants from Roche. FdV reports grants from Foundation STOPbraintumors.org and AbbVIe, BMS, Novartis, EORTC, Vaximm and BioClin Therapeutics. FdV reports participation on a DSMB during the conduct of this study, and leaderships or fiduciary roles in other boards and commissions. All other authors declare no competing interests.

## Appendix Supplementary materials

Image, application 1

Image, application 2
