# Supplementary material for: Efficacy of the eHealth application Oncokompas, facilitating incurably ill cancer patients to self-manage their palliative care needs: A randomized controlled trial
Source: Lancet Reg Health Eur. 2022 Apr 21;18:100390. doi: 10.1016/j.lanepe.2022.100390 (PMC9046636; doi:10.1016/j.lanepe.2022.100390)

**Supplementary material**

**Table of contents**

**Figure 1 page 2**

Overview of the different steps within Oncokompas

**Figure 2 page 4**

Graphics of the course of dyspnea over time for patients participating (partly) before

and (partly) during the COVID-19 pandemic

***Figure 1 – Overview of the different steps within Oncokompas***

| **Step 1: Measure** | **Step 2a: Learn – Overview of well-being on different topics** |
| --- | --- |
| 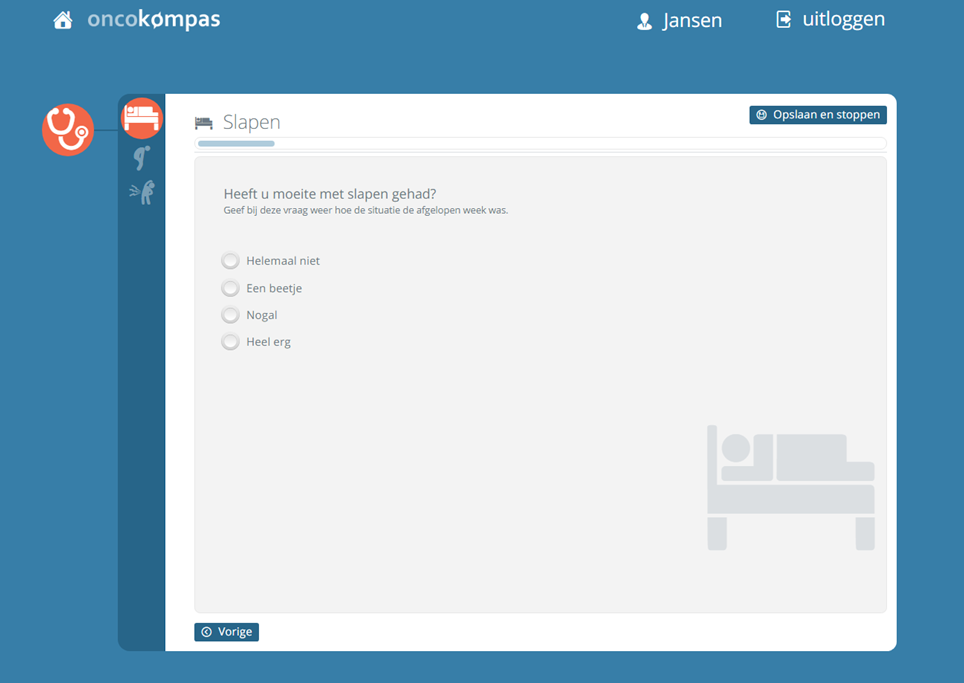 | 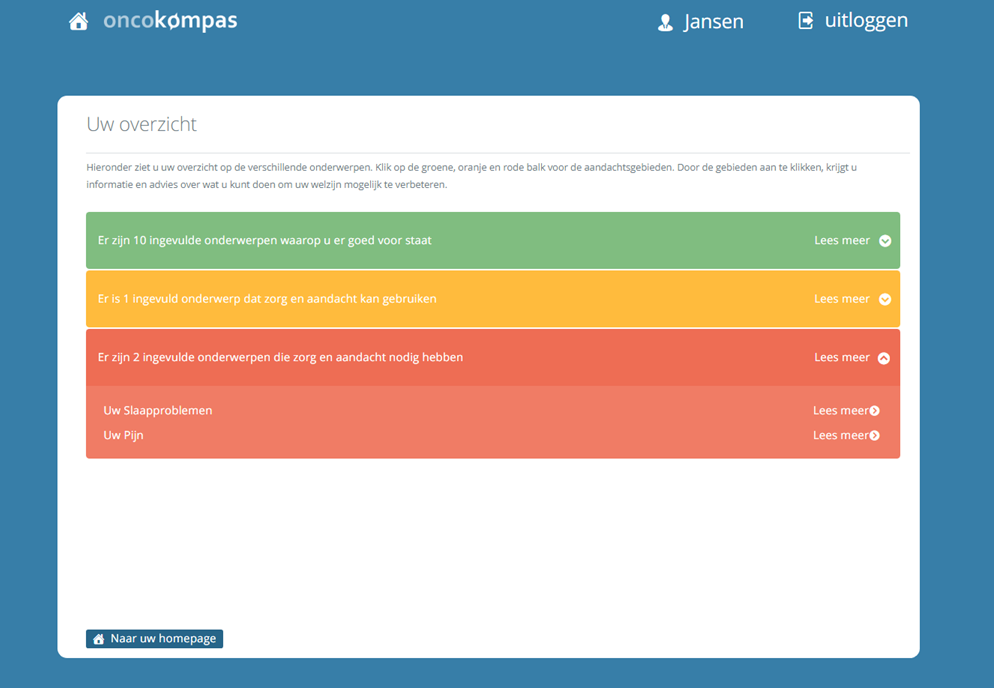 |

| **Step 2b: Learn – Tailored information and advice** | **Step 3: Act** |
| --- | --- |
| 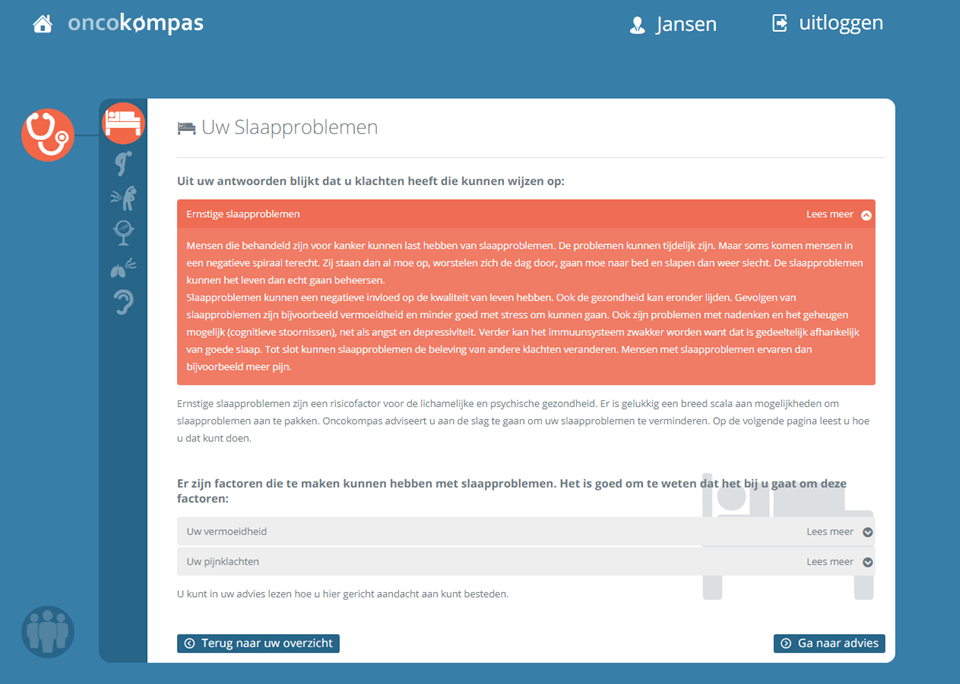 | 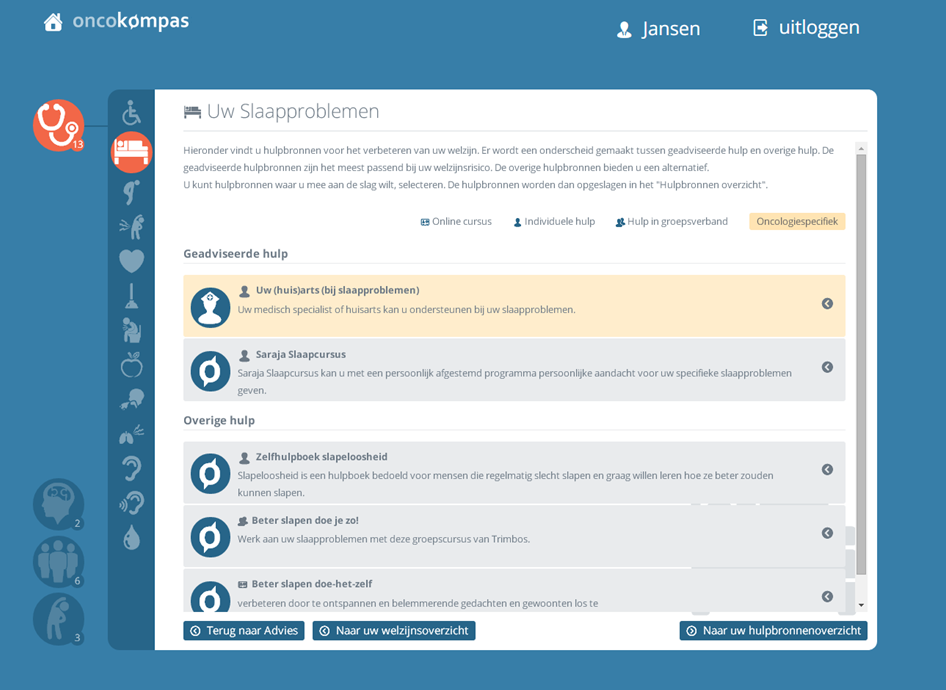 |

***Figure 2 – Graphics of the course of dyspnea over time for patients participating (partly) before and (partly) during the COVID-19 pandemic***


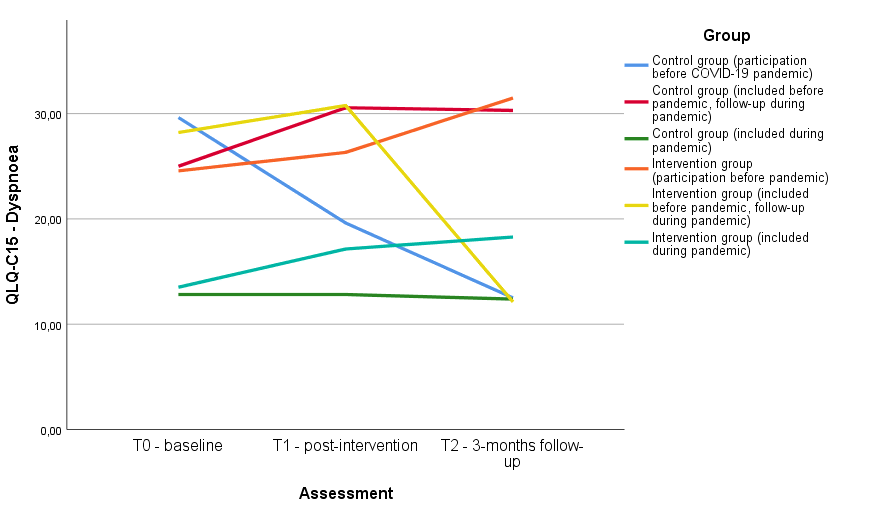

Supplement: Supplementary file 1 [file mmc1.docx]
