# Supplementary material for: Efficacy of the eHealth application Oncokompas, facilitating incurably ill cancer patients to self-manage their palliative care needs: A randomized controlled trial
Source: Lancet Reg Health Eur. 2022 Apr 21;18:100390. doi: 10.1016/j.lanepe.2022.100390 (PMC9046636; doi:10.1016/j.lanepe.2022.100390)
Supplement: Supplementary file 2 [file mmc2.docx]

**Supplementary material**

**Table of contents**

**Table 1 page 2**

Overview of all topics covered in Oncokompas for patients with incurable cancer

**Table 2 page 3**

Mean scores per group per assessment and results of the linear mixed model analyses

on primary and secondary outcome measures for the intervention group

(compliers vs. non-compliers)

**Table 3 page 5**

Mean scores per group per assessment and results of the linear mixed model analyses on

primary and secondary outcome measures for the groups participating before, partly during

and during the COVID-19 pandemic

***Table 1 – Overview of all topics covered in Oncokompas for patients with incurable cancer***

| **Domain** | **Topics** |
| --- | --- |
| Physical | Body weight  Daily functioning  Diarrhea  Dysphagia  Dyspnea  Fatigue  Information about treatment options  Appetite loss  Lymphedema  Mouth problems  Nausea and vomiting  Obstipation  Pain  Sexuality  Skin problems  Sleep problems  Other side effects of medical treatment |
| Psychological | Cancer related anxiety (including fear of suffering and fear of dying)  Coping with emotions  Depression  Tenseness |
| Social | Being single and cancer  Choices concerning the end-of-life  Loneliness  Patient-physician communication  Social life  Relationship with partner  Relationship with (adult) children  Work issues |
| Existential | Meaning of life  Saying farewell |

***Table 2 –*** ***Mean scores per group per assessment and results of the linear mixed model analyses on primary and secondary outcome measures for the intervention group (compliers vs. non-compliers)***

|  |  | Baseline (t0) | | 2 weeks follow-up (t1) | | 3-months follow-up (t2) | |  |  |
| --- | --- | --- | --- | --- | --- | --- | --- | --- | --- |
|  |  | N | Mean (SD) | N | Mean (SD) | N | Mean (SD) | Estimated difference in change between T0 and T2 (90% CI) | P-value two-way interaction |
| Patient activation (PAM) | | | | | | | |  | 0·91 |
|  | Compliers | 45 | 56·6 (12·4) | 46 | 55·8 (13·6) | 41 | 56·9 (11·6) | -1·2 (-5·8 to 3·5) |  |
|  | Non-compliers | 19 | 54·2 (8·7) | 19 | 53·5 (9·4) | 18 | 55·3 (12·2) |  |  |
| General self-efficacy (GSE) | | | | | | | |  | 0·49 |
|  | Compliers | 48 | 29·8 (5·4) | 47 | 29·6 (5·2) | 42 | 30·5 (5·6) | 1·2 (-0·7 to 3·2) |  |
|  | Non-compliers | 20 | 29·4 (5·3) | 20 | 29·3 (6·0) | 18 | 28·8 (5·9) |  |  |
| HRQOL (EORTC-QLQ-C15-PAL) | | | | | | | |  |  |
| Global quality of life | | | | | | | |  | 0·48 |
|  | Compliers | 48 | 72·2 (18·9) | 47 | 69·9 (18·3) | 42 | 70·6 (22·3) | 4·8 (-2·3 to 12·0) |  |
|  | Non-compliers | 20 | 76·7 (18·3) | 20 | 70·0 (18·4) | 18 | 69·4 (19·2) |  |  |
| Physical functioning | | | | | | | |  | 0·28 |
|  | Compliers | 48 | 88·0 (13·9) | 47 | 88·9 (13·1) | 42 | 87·0 (15·3) | 2·3 (-3·0 to 7·7) |  |
|  | Non-compliers | 20 | 90·6 (12·6) | 20 | 86·7 (14·2) | 18 | 87·0 (15·4) |  |  |
| Emotional functioning | | | | | | | |  | 0·33 |
|  | Compliers | 48 | 72·9 (24·9) | 47 | 74·5 (23·3) | 42 | 70·6 (26·2) | -8·2 (-17·4 to 1·0) |  |
|  | Non-compliers | 20 | 69·2 (23·7) | 20 | 75·8 (20·6) | 18 | 75·0 (27·0) |  |  |
| Fatigue | | | | | | | |  | 0·15 |
|  | Compliers | 48 | 44·8 (28·0) | 47 | 44·7 (25·6) | 42 | 42·9 (28·3) | -3·7 (-14·3 to 7·0) |  |
|  | Non-compliers | 20 | 44·2 (26·6) | 20 | 35·8 (21·8) | 18 | 48·1 (29·6) |  |  |
| Pain | | | | | | | |  | 0·85 |
|  | Compliers | 48 | 24·3 (24·5) | 47 | 25·9 (22·2) | 42 | 27·8 (26·5) | 3·5 (-6·8 to 13·7) |  |
|  | Non-compliers | 20 | 33·3 (34·6) | 20 | 32·5 (28·9) | 18 | 33·3 (29·7) |  |  |
| Dyspnea | | | | | | | |  | 0·87 |
|  | Compliers | 48 | 20·1 (23·6) | 47 | 23·4 (26·8) | 42 | 20·6 (25·4) | 2·6 (-12·2 to 7·0) |  |
|  | Non-compliers | 20 | 16·7 (22·9) | 20 | 20·0 (22·7) | 18 | 22·2 (25·6) |  |  |
| Insomnia | | | | | | | |  | 0·92 |
|  | Compliers | 48 | 34·0 (34·0) | 47 | 32·6 (32·2) | 42 | 33·3 (30·4) | 0·6 (-10·0 to 11·2) |  |
|  | Non-compliers | 20 | 31·7 (27·5) | 20 | 28·3 (27·1) | 18 | 29·6 (22·5) |  |  |
| Appetite loss | | | | | | | |  | 0·25 |
|  | Compliers | 48 | 20·1 (28·1) | 47 | 15·6 (28·5) | 42 | 15·9 (23·6) | -11·6 (-23·5 to 0·2) |  |
|  | Non-compliers | 20 | 25·0 (35·7) | 20 | 28·3 (32·9) | 18 | 35·2 (37·0) |  |  |
| Nausea | | | | | | | |  | 0·081 |
|  | Compliers | 48 | 11·8 (20·0) | 47 | 9·2 (15·1) | 42 | 19·8 (27·6) | 18·4 (4·7 to 31·6) |  |
|  | Non-compliers | 20 | 33·3 (41·9) | 20 | 25·0 (35·7) | 18 | 22·2 (30·2) |  |  |
| Constipation | | | | | | | |  | 0·45 |
|  | Compliers | 48 | 21·5 (25·3) | 47 | 20·6 (24·6) | 42 | 21·4 (29·3) | 8·4 (-4·9 to 21·7) |  |
|  | Non-compliers | 20 | 31·7 (31·5) | 20 | 21·7 (29·2) | 18 | 24·1 (19·2) |  |  |

***Table 3 – Mean scores per group per assessment and results of the linear mixed model analyses on primary and secondary outcome measures for the groups participating before, partly during and during the COVID-19 pandemic***

|  |  | Baseline (t0) | | 2 weeks follow-up (t1) | | 3-months follow-up (t2) | |  |  |
| --- | --- | --- | --- | --- | --- | --- | --- | --- | --- |
|  |  | N | Mean (SD) | N | Mean (SD) | N | Mean (SD) | Estimated difference in change between T0 and T2 (90% CI) | P-value three-way interaction |
| Patient activation (PAM) | | | | | | | |  | 0·056 |
| Intervention group | |  |  |  |  |  |  |  |  |
|  | Participation before pandemic* | 18 | 56·7 (13·3) | 19 | 50·0 (9·6) | 18 | 54·8 (11·7) | 0 |  |
|  | Participation partly during pandemic | 12 | 54·5 (13·9) | 13 | 52·3 (16·4) | 11 | 57·2 (15·2) | -1·1 (-9·3 to 7·2) |  |
|  | Participation during pandemic | 35 | 55·4 (9·9) | 33 | 59·2 (11·0) | 30 | 57·1 (10·6) | 6·0 (-0·3 to 12·4) |  |
| Control group | |  |  |  |  |  |  |  |  |
|  | Participation before pandemic | 18 | 52·5 (11·1) | 17 | 51·9 (9·3) | 16 | 52·6 (12·3) |  |  |
|  | Participation partly during pandemic | 12 | 55·5 (11·7) | 12 | 54·7 (8·6) | 11 | 59·7 (8·0) |  |  |
|  | Participation during pandemic | 38 | 56·2 (11·8) | 39 | 56·0 (10·3) | 34 | 54·1 (12·1) |  |  |
| General self-efficacy (GSE) | | | | | | | |  | 0·063 |
| Intervention group | |  |  |  |  |  |  |  |  |
|  | Participation before pandemic | 19 | 29·3 (3·7) | 19 | 29·1 (5·3) | 18 | 28·0 (6·7) | 0 |  |
|  | Participation partly during pandemic | 13 | 28·2 (5·2) | 13 | 27·4 (5·9) | 11 | 29·9 (4·2) | 1·5 (-2·0 to 4·9) |  |
|  | Participation during pandemic | 37 | 30·0 (6·4) | 35 | 30·5 (5·2) | 31 | 31·2 (5·4) | 2·4 (-0·3 to 5·1) |  |
| Control group | |  |  |  |  |  |  |  |  |
|  | Participation before pandemic | 18 | 30·7 (4·5) | 17 | 28·8 (5·9) | 16 | 30·2 (5·1) |  |  |
|  | Participation partly during pandemic | 12 | 30·3 (4·6) | 12 | 31·5 (3·7) | 11 | 31·2 (1·1) |  |  |
|  | Participation during pandemic | 39 | 31·5 (4·6) | 39 | 29·9 (4·3) | 35 | 30·6 (4·5) |  |  |
| HRQOL (EORTC-QLQ-C15-PAL) | | | | | | | |  |  |
| Global quality of life | | | | | | | |  | 0·35 |
| Intervention group | |  |  |  |  |  |  |  |  |
|  | Participation before pandemic | 19 | 70·2 (18·1) | 19 | 64·9 (19·9) | 18 | 64·8 (24·8) | 0 |  |
|  | Participation partly during pandemic | 13 | 66·7 (15·2) | 13 | 66·7 (18·0) | 11 | 65·2 (20·4) | 7·9 (-7·1 to 22·9) |  |
|  | Participation during pandemic | 37 | 78·4 (19·6) | 35 | 73·8 (16·8) | 31 | 75·3 (18·7) | 2·8 (-8·8 to 14·4) |  |
| Control group | |  |  |  |  |  |  |  |  |
|  | Participation before pandemic | 18 | 68·5 (18·0) | 17 | 73·5 (22·9) | 16 | 69·8 (19·5) |  |  |
|  | Participation partly during pandemic | 12 | 72·2 (17·9) | 12 | 72·2 (22·8) | 11 | 68·2 (26·3) |  |  |
|  | Participation during pandemic | 39 | 76·1 (18·7) | 39 | 68·8 (22·7) | 35 | 75·2 (15·8) |  |  |
| Physical functioning | | | | | | | |  | 0·25 |
| Intervention group | |  |  |  |  |  |  |  |  |
|  | Participation before pandemic | 19 | 85·4 (14·8) | 19 | 86·0 (13·3) | 18 | 80·9 (18·6) | 0 |  |
|  | Participation partly during pandemic | 13 | 87·2 (14·2) | 13 | 86·3 (12·1) | 11 | 92·0 (11·2) | 11·3 (0·9 to 21·6) |  |
|  | Participation during pandemic | 37 | 89·8 (14·4) | 35 | 90·2 (13·9) | 31 | 88·9 (13·5) | 5·5 (-2·5 to 13·5) |  |
| Control group | |  |  |  |  |  |  |  |  |
|  | Participation before pandemic | 18 | 91·4 (10·5) | 17 | 87·6 (15·7) | 16 | 91·7 (14·3) |  |  |
|  | Participation partly during pandemic | 12 | 90·7 (10·4) | 12 | 86·1 (15·8) | 11 | 86·9 (13·0) |  |  |
|  | Participation during pandemic | 39 | 89·7 (19·8) | 39 | 88·0 (20·6) | 35 | 87·9 (21·5) |  |  |
| Emotional functioning | | | | | | | |  | 0·41 |
| Intervention group | |  |  |  |  |  |  |  |  |
|  | Participation before pandemic | 19 | 66·7 (22·9) | 19 | 69·3 (23·1) | 18 | 65·7 (31·0) | 0 |  |
|  | Participation partly during pandemic | 13 | 65·4 (30·0) | 13 | 67·9 (29·2) | 11 | 62·1 (28·0) | 1·4 (-15·4 to 18·2) |  |
|  | Participation during pandemic | 37 | 76·6 (22·4) | 35 | 80·5 (17·8) | 31 | 79·0 (21·1) | 9·6 (-3·5 to 22·6) |  |
| Control group | |  |  |  |  |  |  |  |  |
|  | Participation before pandemic | 18 | 75·9 (23·7) | 17 | 79·4 (22·5) | 16 | 79·2 (24·0) |  |  |
|  | Participation partly during pandemic | 12 | 77·8 (25·9) | 12 | 69·4 (24·4) | 11 | 72·7 (29·1) |  |  |
|  | Participation during pandemic | 39 | 84·6 (17·7) | 39 | 85·5 (17·6) | 35 | 78·6 (19·2) |  |  |
| Fatigue | | | | | | | |  | 0·31 |
| Intervention group | |  |  |  |  |  |  |  |  |
|  | Participation before pandemic | 19 | 49·1 (30·7) | 19 | 44·7 (23·6) | 18 | 52·8 (28·7) | 0 |  |
|  | Participation partly during pandemic | 13 | 52·3 (24·4) | 13 | 47·4 (22·4) | 11 | 43·9 (28·2) | -23·0 (-43·1 to 2·9) |  |
|  | Participation during pandemic | 37 | 41·0 (27·7) | 35 | 38·6 (26·1) | 31 | 39·8 (28·4) | -12·3 (-27·9 to 3·3) |  |
| Control group | |  |  |  |  |  |  |  |  |
|  | Participation before pandemic | 18 | 39·8 (30·9) | 17 | 39·2 (35·8) | 16 | 30·2 (27·4) |  |  |
|  | Participation partly during pandemic | 12 | 29·2 (28·5) | 12 | 38·9 (32·8) | 11 | 39·4 (35·2) |  |  |
|  | Participation during pandemic | 39 | 35·9 (22·1) | 39 | 38·9 (27·4) | 35 | 41·0 (25·4) |  |  |
| Pain | | | | | | | |  | 0·29 |
| Intervention group | |  |  |  |  |  |  |  |  |
|  | Participation before pandemic | 19 | 35·1 (32·8) | 19 | 24·6 (24·4) | 18 | 31·5 (31·8) | 0 |  |
|  | Participation partly during pandemic | 13 | 29·5 (20·6) | 13 | 39·7 (22·1) | 11 | 28·8 (23·7) | 5·1 (-13·8 to 23·9) |  |
|  | Participation during pandemic | 37 | 23·0 (27·6) | 35 | 25·2 (24·4) | 31 | 28·5 (26·6) | -1·6 (-16·2 to 13·0) |  |
| Control group | |  |  |  |  |  |  |  |  |
|  | Participation before pandemic | 18 | 21·3 (26·1) | 17 | 21·6 (24·8) | 16 | 18·8 (24·2) |  |  |
|  | Participation partly during pandemic | 12 | 29·2 (25·7) | 12 | 26·4 (30·5) | 11 | 24·2 (20·2) |  |  |
|  | Participation during pandemic | 39 | 21·8 (25·1) | 39 | 26·5 (26·4) | 35 | 26·7 (30·3) |  |  |
| Dyspnea | | | | | | | |  | 0·018 |
| Intervention group | |  |  |  |  |  |  |  |  |
|  | Participation before pandemic | 19 | 24·6 (24·4) | 19 | 26·3 (30·6) | 18 | 31·5 (33·3) | 0 |  |
|  | Participation partly during pandemic | 13 | 28·2 (26·7) | 13 | 30·8 (25·3) | 11 | 12·1 (16·8) | -36·7 (-54·2 to -19·2) |  |
|  | Participation during pandemic | 37 | 13·5 (20·0) | 35 | 17·1 (21·9) | 31 | 18·3 (20·8) | -14·0 (-27·5 to -0·5) |  |
| Control group | |  |  |  |  |  |  |  |  |
|  | Participation before pandemic | 18 | 29·6 (37·7) | 17 | 19·6 (35·5) | 16 | 12·5 (26·9) |  |  |
|  | Participation partly during pandemic | 12 | 25·0 (20·7) | 12 | 30·6 (30·0) | 11 | 30·3 (31·5) |  |  |
|  | Participation during pandemic | 39 | 12·8 (18·1) | 39 | 12·8 (19·7) | 35 | 12·4 (21·5) |  |  |
| Insomnia | | | | | | | |  | 0·14 |
| Intervention group | |  |  |  |  |  |  |  |  |
|  | Participation before pandemic | 19 | 33·3 (27·2) | 19 | 31·6 (28·3) | 18 | 33·3 (25·6) | 0 |  |
|  | Participation partly during pandemic | 13 | 46·2 (39·8) | 13 | 46·2 (39·8) | 11 | 30·3 (34·8) | -9·4 (-31·8 to 13·0) |  |
|  | Participation during pandemic | 37 | 28·8 (30·6) | 35 | 25·7 (26·9) | 31 | 32·3 (27·9) | 5·4 (-11·9 to 22·7) |  |
| Control group | |  |  |  |  |  |  |  |  |
|  | Participation before pandemic | 18 | 37·0 (30·0) | 17 | 27·5 (35·8) | 16 | 33·3 (32·2) |  |  |
|  | Participation partly during pandemic | 12 | 36·1 (38·8) | 12 | 30·6 (26·4) | 11 | 27·3 (32·7) |  |  |
|  | Participation during pandemic | 39 | 23·9 (26·4) | 39 | 29·1 (26·7) | 35 | 21·9 (27·9) |  |  |
| Appetite loss | | | | | | | |  | 0·94 |
| Intervention group | |  |  |  |  |  |  |  |  |
|  | Participation before pandemic | 19 | 29·8 (38·3) | 19 | 28·1 (37·3) | 18 | 25·9 (35·3) | 0 |  |
|  | Participation partly during pandemic | 13 | 17·9 (22·0) | 13 | 20·5 (25·6) | 11 | 15·2 (22·9) | -0·5 (-24·4 to 23·5) |  |
|  | Participation during pandemic | 37 | 19·8 (28·8) | 35 | 14·3 (27·2) | 31 | 21·5 (28·0) | -6·3 (-24·8 to 12·2) |  |
| Control group | |  |  |  |  |  |  |  |  |
|  | Participation before pandemic | 18 | 31·5 (31·3) | 17 | 29·4 (37·0) | 16 | 25·0 (31·0) |  |  |
|  | Participation before pandemic | 12 | 16·7 (22·5) | 12 | 19·4 (30·0) | 11 | 18·2 (27·3) |  |  |
|  | Participation partly during pandemic | 39 | 12·8 (23·7) | 39 | 16·2 (25·2) | 35 | 21·9 (30·2) |  |  |
| Nausea | | | | | | | |  | 0·55 |
| Intervention group | |  |  |  |  |  |  |  |  |
|  | Participation before pandemic | 19 | 29·8 (36·7) | 19 | 17·5 (28·0) | 18 | 25·9 (35·3) | 0 |  |
|  | Participation partly during pandemic | 13 | 10·3 (25·0) | 13 | 10·3 (16·0) | 11 | 12·1 (22·5) | 20·1 (-5·4 to 45·7) |  |
|  | Participation during pandemic | 37 | 16·2 (26·8) | 35 | 13·3 (24·5) | 31 | 20·4 (25·4) | 13·6 (-6·1 to 33·4) |  |
| Control group | |  |  |  |  |  |  |  |  |
|  | Participation before pandemic | 18 | 16·7 (23·6) | 17 | 17·6 (35·6) | 16 | 20·8 (29·5) |  |  |
|  | Participation partly during pandemic | 12 | 19·4 (33·2) | 12 | 11·1 (21·7) | 11 | 12·1 (22·5) |  |  |
|  | Participation during pandemic | 39 | 19·7 (22·6) | 39 | 16·2 (25·2) | 35 | 19·0 (27·2) |  |  |
| Constipation | | | | | | | |  | 0·87 |
| Intervention group | |  |  |  |  |  |  |  |  |
|  | Participation before pandemic | 19 | 31·6 (30·4) | 19 | 21·1 (29·8) | 18 | 27·8 (32·8) | 0 |  |
|  | Participation partly during pandemic | 13 | 10·3 (21·0) | 13 | 20·5 (29·0) | 11 | 12·1 (22·5) | 2·4 (-22·2 to 27·1) |  |
|  | Participation during pandemic | 37 | 25·2 (26·5) | 35 | 21·0 (23·0) | 31 | 22·6 (23·4) | -6·6 (-25·6 to 12·5) |  |
| Control group | |  |  |  |  |  |  |  |  |
|  | Participation before pandemic | 18 | 20·4 (23·3) | 17 | 17·6 (31·4) | 11 | 18·8 (27·1) |  |  |
|  | Participation partly during pandemic | 12 | 16·7 (17·4) | 12 | 27·8 (31·2) | 16 | 21·2 (30·8) |  |  |
|  | Participation during pandemic | 39 | 18·8 (22·7) | 39 | 17·1 (25·2) | 35 | 22·9 (30·0) |  |  |

* Pandemic = COVID-19 pandemic
